# Simulation of GO–PAMAM-Modified Polysulfone Substrate-Based Thin-Film Composite Reverse-Osmosis Membranes for Desalination

**DOI:** 10.3390/membranes16060184

**Published:** 2026-05-28

**Authors:** Mohd Muzammil Zubair, Syed Javaid Zaidi

**Affiliations:** 1 UNESCO Chair in Desalination and Water Treatment, Center for Advanced Material, Qatar University, Doha P.O. Box 2713, Qatar; 2 Department of Mechanical and Industrial Engineering, Qatar University, Doha P.O. Box 2713, Qatar

**Keywords:** reverse osmosis (RO), thin-film composite (TFC), computational fluid dynamics (CFD), polysulfone substrate, salt rejection

## Abstract

Freshwater scarcity driven by population growth and industrial demand has increased reliance on desalination, where reverse osmosis (RO) is widely applied due to its high separation efficiency. Membrane performance is governed by the balance between water permeability and solute rejection, and attempts to improve this relationship have focused on incorporating nanomaterials to modify membrane structure and transport behavior. In this study, a computational investigation was carried out for thin-film composite (TFC) membranes incorporating graphene oxide–poly(amidoamine) (GO–PAMAM) within the polysulfone substrate to examine its influence on transport under RO conditions. A two-dimensional model was implemented in COMSOL Multiphysics by coupling the Laminar Flow and Transport of Diluted Species interfaces, while permeation across the membrane was described using a solution–diffusion framework parameterized by experimentally determined salt permeability coefficient. Variation in GO–PAMAM loading (0–0.10 wt%) was introduced through intrinsic permeability parameters, enabling direct comparison with experimental data. The simulations reproduced the observed trends, with the membrane containing 0.06 wt% GO–PAMAM showing higher salt rejection, increasing from 78.16% to 90.08% relative to the pristine membrane. The model predicted lower permeate-side solute concentration and a decrease in salt rejection along the membrane length. Model predictions agreed with experiments, with mean relative errors of 1.23% for salt rejection and 7.41% for water flux, demonstrating the ability of the model to capture transport behavior in GO–PAMAM-modified TFC membranes.

## 1. Introduction

Freshwater scarcity is increasing due to population growth, industrial activity, urbanization, and climate-related pressure on conventional water resources [1]. Desalination is therefore an important approach for augmenting water supplies from saline and brackish sources [2]. Among desalination technologies, reverse osmosis (RO) is widely used because of its high separation efficiency, modular operation, and lower energy demand compared with thermal desalination processes [3]. However, RO membrane performance remains limited by the permeability–selectivity trade-off, in which higher water permeability can be accompanied by increased salt passage or reduced membrane stability. Improving this balance requires membrane structures that enhance water transport while maintaining selective salt rejection [4]. Thin-film composite (TFC) membranes are the main architecture used in RO applications. They consist of an ultrathin polyamide (PA) selective layer formed on a porous polymeric support, commonly polysulfone (PSF), with a nonwoven polyester backing layer [5,6,7] Although the PA layer is primarily responsible for salt rejection, its formation is strongly influenced by the properties of the underlying support. Support wettability, porosity, pore size distribution, roughness, and surface chemistry affect aqueous monomer uptake and diffusion during interfacial polymerization, which, in turn, influence PA thickness, crosslinking, and defect formation [8,9]. Therefore, support layer modification can improve TFC membrane performance without directly changing the PA chemistry [10].

Nanomaterials have been widely incorporated into the PA selective layer to form thin-film nanocomposite (TFN) membranes, with the aim of improving hydrophilicity and regulating water and solute transport pathways [11]. Reported TFN membranes based on metal oxides, carbon-based nanomaterials, zeolitic frameworks, and dendrimer-functionalized fillers have shown that nanoscale additives can improve water permeation and salt rejection when they are well dispersed within the selective layer. However, direct nanoparticle incorporation into the PA layer can also lead to aggregation, poor interfacial compatibility, and nonselective void formation, which may reduce salt rejection and affect membrane stability [12]. These limitations have shifted attention toward substrate-level modification, where nanoparticles are introduced into the PSF support to control support morphology, monomer uptake, and PA layer formation during interfacial polymerization.

To regulate IP through substrate engineering, the support modifier must enhance surface hydrophilicity, interact favorably with the polymer matrix, and remain well dispersed during phase inversion. Two-dimensional nanomaterials are attractive in this regard because their high aspect ratio enables effective surface modification at low loading.

Following this approach, substrate modification using nanoparticles has been investigated in forward osmosis (FO), nanofiltration (NF), and RO membranes to regulate IP and enhance separation performance.

Emadzadeh et al. incorporated TiO_2_ nanoparticles into the PSF support layer to fabricate TFC membranes and evaluate their performance under FO [13]. TiO_2_ nanoparticles were added at four different concentrations (0, 0.50, 0.75, and 0.99 wt%), among which the membrane consisting of 0.50 wt% of TiO_2_ in the PSF matrix outperformed the other membranes in terms of higher water permeability and lower reverse solute flux. The addition of nanoparticles also contributed to improving the hydrophilicity and porosity of the membranes.

NF salt rejection studies were carried out by Mohammed et al. [14]. using salts such as NaCl, MgCl_2_, MgSO_4_, and Na_2_SO_4_ by incorporating nanozeolite into the PVP + PSF solution matrix at a concentration ranging from 0 to 0.2 wt%. The PA layer was formed by using reagents such as piperazine (PIP) and trimesoyl chloride (TMC) at concentrations of 2 wt% in water and 0.25 wt% in hexane, respectively. They also concluded that the addition of nanoparticles into the substrate influenced the porosity, wettability, and pore size of the membrane. Optimum results were obtained at 0.1 wt%, with pure water permeability of 17.1 ± 2.1 LMH/bar and a maximum salt rejection of 95.1% for Na_2_SO_4_.

Panahi et al. [15] investigated an RO system by developing TFC membranes through the incorporation of GO/SiO_2_ nanoparticles into the substrate layer at four different concentrations while keeping the PA selective layer pristine in all membranes. The highest salt rejection for different salts, including NaCl, MgCl_2_, CaCl_2_, and Na_2_SO_4_, was achieved by the membrane containing 0.5 wt% nanoparticles, with the maximum rejection observed for Na_2_SO_4_ (97.02%). In terms of water flux, the membrane containing 1.0 wt% nanoparticles in the substrate outperformed the other membranes; however, this improvement was accompanied by a reduction in salt rejection efficiency. Their study also showed that nanoparticle incorporation decreased membrane hydrophobicity, as indicated by a 25% reduction in contact angle.

Graphene oxide (GO) is widely studied due to its oxygen-containing functional groups, which improve hydrophilicity and compatibility with polar polymers and influence pore formation during phase inversion [16]. However, pristine GO may agglomerate within polymer matrices, limiting its effectiveness. Furthermore, poly(amidoamine) (PAMAM) dendrimers offer an abundance of amine functional groups that can augment wettability and improve interactions within the polymer matrix [17]. GO functionalized with PAMAM (GO–PAMAM) integrates these benefits by enhancing dispersibility and facilitating robust interactions with the polymer solution during phase inversion [18]. In our previous experimental study, FTIR analysis confirmed the successful incorporation of GO–PAMAM within the polysulfone matrix, while contact angle measurements further supported the improvement in substrate hydrophilicity through a reduction in contact angle [19]. When integrated into PSF supports, GO–PAMAM is anticipated to alter pore formation and enhance substrate hydrophilicity, thereby affecting monomer distribution during interfacial polymerization and indirectly facilitating PA layer development. The performance improvement in this approach does not arise from direct molecular sieving by the support layer. Instead, the modified substrate influences the formation conditions of the PA selective layer, indirectly improving its structural quality and transport characteristics.

Although experimental characterization can confirm GO–PAMAM incorporation and its effect on substrate wettability, it cannot fully describe how these substrate-level changes influence local flow behavior, solute distribution, and membrane transport under operating RO conditions. Despite these advances, the relationship between substrate modification and membrane transport behavior remains insufficiently resolved, particularly under operating conditions where flow and mass transfer are coupled. Experimental studies primarily report overall performance metrics such as water flux and salt rejection, but they provide limited insight into the spatial variation of velocity and solute concentration within the membrane module. In this context, CFD modeling provides a framework to examine the governing transport phenomena and relate intrinsic membrane properties to observed performance. In this study, COMSOL Multiphysics was employed to simulate the reverse osmosis process for thin-film composite membranes incorporating GO–PAMAM within the polysulfone substrate. A two-dimensional model was developed by coupling the Laminar Flow and Transport of Diluted Species interfaces, while membrane transport was described using a solution–diffusion formulation parameterized by experimentally determined intrinsic salt permeability coefficient. The model was used to evaluate water flux and salt rejection at different GO–PAMAM loadings and examine the influence of substrate modification on transport behavior along the membrane length. The novelty of this work lies in linking GO–PAMAM-induced substrate modification with membrane-scale transport behavior using a CFD model parameterized by experimentally determined water and salt permeability coefficients. This approach differs from previous TFC membrane studies that primarily focused on experimental performance evaluation, as it enables visualization of local velocity and solute concentration distributions and provides mechanistic insight into how substrate modification affects RO performance. The predicted results are compared with experimental data to assess model accuracy and provide insight into the relationship between substrate structure and membrane performance.

## 2. Experimental Procedure

### Membrane Preparation

GO was synthesized from graphite powder using a modified Hummers’ method, as reported by Mahmoudi et al. [20]. After synthesis, the GO was repeatedly washed, purified, and dried prior to functionalization. GO–PAMAM nanocomposites were prepared by grafting PAMAM dendrimers onto GO sheets [21]. To facilitate sufficient exfoliation, GO was dispersed in *N,N*-Dimethylformamide (DMF) with continuous stirring, whereas PAMAM dendrimers were dissolved separately in methanol. Subsequently, the PAMAM solution was introduced dropwise into the GO dispersion, and the resulting mixture underwent refluxing at an elevated temperature to encourage functionalization. After completion of the reaction, the product was washed thoroughly with ethanol and distilled water to remove unreacted species and then dried to obtain GO–PAMAM nanocomposite powder. The constructive materials of the modified membrane, along with their specifications, roles, and applications, are summarized in Table 1.

TFC membranes were fabricated using a combination of non-solvent-induced phase separation and IP. Porous PSF support membranes were prepared by dissolving PSF in DMF to obtain a casting solution containing 17.5 wt% PSF. Predetermined amounts of GO–PAMAM nanocomposite (0.03, 0.06, and 0.10 wt%) were first dispersed in DMF by ultrasonication, after which PSF granules were gradually added under stirring at 60 °C until complete dissolution. The resulting dope solution was then allowed to stand for 4 h to remove entrapped air bubbles. The prepared casting solution was spread onto a nonwoven polyester fabric using a casting knife with a 200 µm gap and immediately immersed in a coagulation bath at ambient temperature. Phase inversion induced the formation of an asymmetric porous PSF support layer firmly bonded to the backing fabric. The resulting membranes were kept in distilled water for 24 h to remove residual solvent and stabilize the pore structure. The PA selective layer was formed on the prepared PSF supports via interfacial polymerization. The aqueous phase consisted of a 3 wt% m-phenylenediamine (MPD) solution containing 0.2 wt% sodium dodecyl sulfate as a surfactant. The support membrane was immersed in the aqueous solution, and any surplus solution was eliminated using a rubber roller after 4 min. Following this, a 0.1 wt% TMC solution in n-hexane was introduced for a brief reaction period to initiate the formation of the PA layer. Following this, the membranes were thermally cured at 70 °C for a duration of ten minutes, subsequently rinsed thoroughly with deionized water, and then stored prior to characterization and performance evaluation.

The membranes were categorized as M0 (pristine), M1 (0.03 wt%), M2 (0.06 wt%), and M3 (0.10 wt%), contingent upon the GO–PAMAM loading within the PSF substrate.

## 3. System Specification

This study examined how adding GO–PAMAM to the support layer affected the performance of the TFC membranes used in the RO separation process. The numerical model was structured around two interconnected flow domains: a feed channel, which handled the incoming saline solution, and a permeate channel, which was responsible for the collection of the permeate. The feed solution entered the feed channel through the inlet boundary, flowed along the membrane surface, and any portion of the feed that did not permeate exited through the feed outlet. Water permeated across the TFC membrane, which was represented in the model as an internal semi-permeable interface separating the feed and permeate channels, and subsequently entered the permeate channel, where it exited through the permeate outlet. Within the simulation framework, the membrane was conceptualized as an effective transport barrier, its function dictated by the solution–diffusion transport mechanism; this allowed for water permeation while simultaneously impeding salt transport. The principal aim of utilizing CFD modeling was to investigate the impact of substrate modification, specifically with varying GO–PAMAM loadings, on membrane transport characteristics, with a focus on water flux and salt rejection, under reverse osmosis RO operating conditions. The simulations were run using COMSOL Multiphysics. This software uses the finite element method to solve related partial differential equations that describe laminar flow and how solutes move.

**Figure 1:**
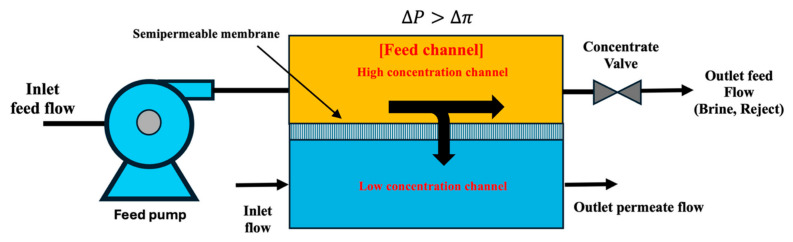
Schematic diagram of the reverse osmosis process showing the feed and permeate channels separated by a semi-permeable membrane under an applied pressure gradient (ΔP > Δπ).

The Laminar Flow and Transport of Diluted Species (TDS) physics interfaces were used to model incompressible flow and convective–diffusive salt transport in both feed and permeate channels. The applied hydraulic pressure on the feed side exceeded the osmotic pressure difference across the membrane, resulting in water transport from the high-salinity feed side to the low-salinity permeate side, opposite to natural osmosis. A schematic representation of the RO system and computational domain is shown in Figure 1, while the main geometric, operating, and transport parameters used in the simulation are summarized in Table 2.

The feed NaCl concentration used in the model was 34.2 mol m^−3^, corresponding to 2000 mg L^−1^ NaCl, which was selected to match the experimental membrane testing condition. This concentration is lower than typical seawater salinity and was used here to evaluate and compare the intrinsic transport behavior of the fabricated membranes under controlled laboratory conditions. Therefore, the present simulation should be interpreted as validation under the tested low-salinity/brackish-type feed condition rather than as a complete seawater RO prediction. Future work will extend the model to higher feed salinities representative of seawater desalination.

The modified TFC membranes were represented in the model by assigning different intrinsic transport parameters to the membrane interface. The effect of GO–PAMAM incorporation in the support layer was introduced through experimentally determined salt permeability (B) coefficients corresponding to each membrane. Based on the GO–PAMAM loading in the PSF substrate, the membranes were designated as M0 (pristine), M1, M2, and M3 in increasing order of nanocomposite content. The specific transport properties of each membrane used as model inputs are listed in Table 3.

## 4. Numerical Modeling

This research modeled the RO membrane process using modified TFC membranes. These membranes included GO–PAMAM in the support layer. The impact of different GO–PAMAM loadings in the membrane substrate on water flux and salt rejection was studied using COMSOL Multiphysics. Modeling the RO system required solving the governing equations that described fluid flow and solute transport simultaneously. This was effectively done using the multiphysics capabilities of COMSOL Multiphysics software.

This investigation primarily employed the continuity and Navier–Stokes equations, which governed laminar fluid flow, and the convection–diffusion equation, which characterized solute transport within both the feed and permeate channels. The Laminar Flow and Transport of Diluted Species physics interfaces within COMSOL Multiphysics were employed to address these coupled transport phenomena.

Prior research has demonstrated that three-dimensional flow and mass transfer visualizations offer a comprehensive understanding of transport phenomena in RO systems [22]. Nevertheless, the precise forecasting of concentration gradients adjacent to the membrane surface often necessitates highly refined computational grids, which, in turn, elevates computational expenses and prolongs solution durations [23]. Consequently, a two-dimensional model could represent a viable balance between computational economy and predictive fidelity. Furthermore, the application of two-dimensional calculations facilitates the efficient examination of how modeling assumptions and numerical parameters influence membrane performance, especially within channel-based RO designs [24].

In this study, a two-dimensional computational domain was utilized to model the RO process. Given the membrane’s comparatively small thickness relative to its length and width, a two-dimensional representation offered a suitable approximation of transport dynamics, thereby substantially decreasing the computational time needed for numerical solutions. Consequently, this methodology facilitated an efficient examination of how GO–PAMAM substrate modification influences intrinsic membrane transport characteristics within the context of RO operating conditions.

### 4.1. Assumptions

The following assumptions were considered in the present model:

- The flow in the feed and permeate channels was assumed to be laminar [25].
- The TFC membrane was represented as a semi-permeable internal interface.
- Water and solute transport across the membrane followed the solution–diffusion mechanism.
- The influence of GO–PAMAM incorporation in the support layer was introduced through the intrinsic membrane transport parameter, namely, salt permeability (B).
- The system was assumed to operate under steady-state and isothermal conditions [26].

### 4.2. Fluid Flow Modeling

The hydrodynamic characteristics of the feed solution within the RO system were modeled, drawing upon the geometry of a laboratory-scale crossflow filtration unit, specifically the Sterlitech CF042 (Auburn, WA, USA) membrane test cell. This membrane cell possessed dimensions of 9.2 cm × 4.6 cm × 0.23 cm (length × width × height), which corresponded to an effective membrane filtration area of roughly 42 cm^2^. For the purpose of assessing membrane performance, a standard aqueous sodium chloride (NaCl) solution, prepared at a concentration of 2000 ppm, served as the feed solution.

The Reynolds number within the feed channel was calculated according to

(1) $$Re=\frac{\rho vd}{\mu }$$

where *ρ* is the density of the solution (kg m^−3^), *v* is the cross-flow velocity (m s^−1^), *μ* is the dynamic viscosity (Pa s), and *d* is the characteristic length. In the present study, the channel height was used as the characteristic length based on the parallel-plate approximation for rectangular membrane channels with large aspect ratios [27].

For the operating condition corresponding to a feed flow rate of 3 LPM, the inlet velocity in the feed channel was calculated to be approximately 0.56 m s^−1^, resulting in a Reynolds number of 1442. Since the flow remained within the laminar regime in confined non-circular membrane channels, laminar flow conditions were assumed throughout the computational domain. All simulations were performed under steady-state and isothermal conditions at 25 °C, and the physical properties of the feed solution were assumed to remain constant throughout the simulation domain.

The fluid flow was modeled using the Laminar Flow interface in COMSOL Multiphysics, which solves the continuity equation for mass conservation and the Navier–Stokes equations for momentum conservation for an incompressible Newtonian fluid. The governing equations describing fluid motion in both feed and permeate channels were given as

(2) $$\frac{\partial \rho }{\partial t}+\nabla \cdot \left(\rho U\right)=0$$

(3) $$\frac{\partial \left(\rho U\right)}{\partial t}+\nabla \left(\rho UU\right)=\nabla \left[\mu \left(\nabla U+\nabla U\nabla^{T}\right)\right]-\nabla P+\rho g$$

To ensure numerical stability and eliminate pressure indeterminacy in the permeate channel, a secondary auxiliary inlet stream with negligible velocity and solute concentration was introduced into the permeate domain. This auxiliary stream was implemented solely to provide a pressure reference and facilitate solver convergence without affecting the predicted hydrodynamic behavior.

At the feed inlet, a uniform velocity boundary condition corresponding to the specified crossflow rate was applied, whereas the outlet boundaries of both feed and permeate channels were defined using pressure outlet conditions with suppressed backflow. No-slip boundary conditions were imposed on all channel walls and membrane interfaces. A schematic representation of the RO system and the two-dimensional computational domain, including the feed and permeate channels separated by the semipermeable membrane, is shown in Figure 1, while the main geometric and operating parameters used in the simulation are summarized in Table 2.

### 4.3. Mass Transfer Modeling

The Transport of Diluted Species (TDS) module available in COMSOL Multiphysics was utilized to simulate the transport of dissolved salt through the feed and permeate channels and determine the passage or rejection of salt across the TFC-RO membrane. The transport of species within the computational domain is governed by the combined effects of convection and diffusion. The governing equations describing the mass transport of solute are expressed as follows:

(4) $$\nabla J_{i}+U\cdot \nabla C_{i}=R_{i}$$

(5) $$J_{i}=D_{i}\nabla C_{i}$$

(6) $$\frac{\partial C_{i}}{\partial t}+\nabla J_{i}+U\cdot \nabla C_{i}=0$$

where $J_{i}$ represents the molar flux of species (mol m^−2^ s^−1^), $C_{i}$ denotes the concentration of the dissolved species (mol m^−3^), U is the velocity vector (m s^−1^) obtained from the laminar flow interface, and $R_{i}$ represents the rate of generation or consumption of species (mol m^−3^ s^−1^). The parameter $D_{i}$ in Equation (5) corresponds to the diffusion coefficient of the solute in the aqueous medium (m^2^ s^−1^).

In this study, the boundary between the feed and permeate channels representing the TFC membrane was modeled as an interior semi-permeable interface allowing selective transport of species. The mass flux across this membrane boundary was defined using a flux condition expressed as

(7) $$-n\cdot \left(-D_{i}\nabla C_{i}\right)=J_{0}$$

where *n* represents the outward normal vector to the membrane boundary and $J_{0}$ denotes the prescribed flux of the species across the membrane interface. At the feed inlet boundary, the concentration of dissolved salt was specified based on the experimental operating condition used for membrane testing. An aqueous sodium chloride solution of 2000 ppm, corresponding to a molar concentration of 34.2 mol m^−3^, was considered as the input feed solution and defined as both the initial and inlet concentration of the species within the feed channel. At the outlet boundaries of the feed and permeate domains, an outflow boundary condition was applied assuming convection-dominated transport, and, therefore, the diffusive flux normal to the boundary was neglected, which was given by

(8) $$n\cdot \left(-D_{i}\nabla C_{i}\right)=0$$

Finally, the salt rejection performance of the GO–PAMAM-modified TFC membranes was evaluated using the simulated feed and permeate concentrations obtained from the numerical model. The percentage of salt rejection was calculated as follows:

(9) $$\%R=\left(1-\frac{C_{p}}{C_{f}}\right)\times 100$$

where *C_f_* and *C_p_* represent the salt concentrations in the feed and permeate streams (mol m^−3^), respectively.

In the present model, pressure-driven water permeation was not explicitly solved using a hydraulic permeability expression. Salt transport was represented using the membrane-specific salt permeability coefficient, *B*, and salt rejection was calculated from the simulated feed and permeate concentrations. The water flux was subsequently estimated from the calculated rejection and salt permeability coefficient using Equation (10). Therefore, the effects of varying feed salinity and pressure difference were not investigated parametrically in this study.

(10) $$J=\frac{B\cdot R}{1-R}$$

### 4.4. Mesh Structure

Governing transport equations that describe fluid flow and solute mass transfer were numerically solved using the finite element method (FEM). In this method, the entire geometry was discretized into a finite number of non-overlapped connected meshes, thereby deriving simplified algebraic equations from partial differential equations that governed the fluid flow and solute transport [28,29]. The field variables, including velocity, pressure, and solute concentration, were approximated in each element using low-order polynomial interpolation functions. These individual approximations were then combined to obtain a continuous solution across the entire computational domain.

**Figure 2:**
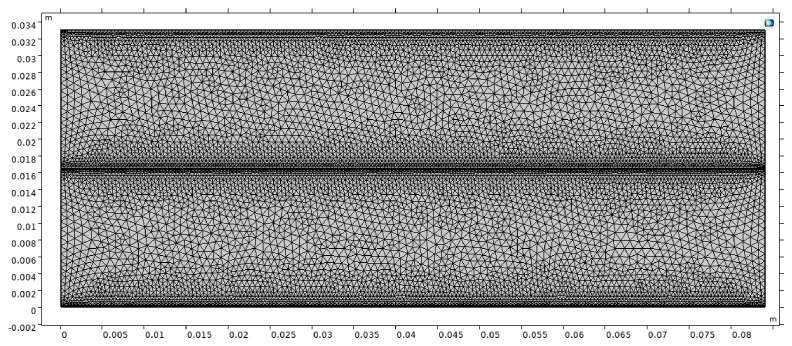
Mesh distribution of the computational domain representing the feed and permeate channels separated by the semi-permeable membrane.

The mesh distribution of the developed two-dimensional computational domain is shown in Figure 2. The two-dimensional meshing of geometry was executed utilizing the physics-controlled mesh within COMSOL Multiphysics, which dynamically adjusted the grid based on the dominant transport processes. A mesh independence test was also carried out to make sure that further refinement of the mesh did not significantly affect the simulation results. Four progressively refined mesh configurations (normal, fine, finer, and extra fine) were evaluated by comparing the predicted membrane performance parameters, including permeate concentration and salt rejection. It was observed that the transition from a normal to a finer mesh significantly improved numerical accuracy, whereas further refinement beyond the finer mesh level resulted in negligible variation in the simulation outcomes. This confirms that the adopted mesh configuration provided a grid-independent solution while maintaining computational efficiency. The final discretized domain of the developed two-dimensional model consisted of a total of 17,866 finite elements.

All geometric parameters and feed solution concentration values used in this simulation study were defined based on the experimentally fabricated TFC membranes developed in the previous work [19].

### 4.5. Model Validation

**Figure 3:**
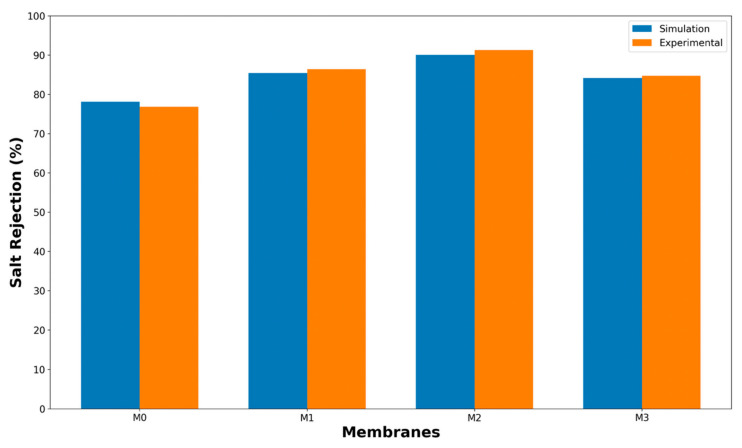
Comparison of simulation and experimental salt rejection for different membranes.

**Figure 4:**
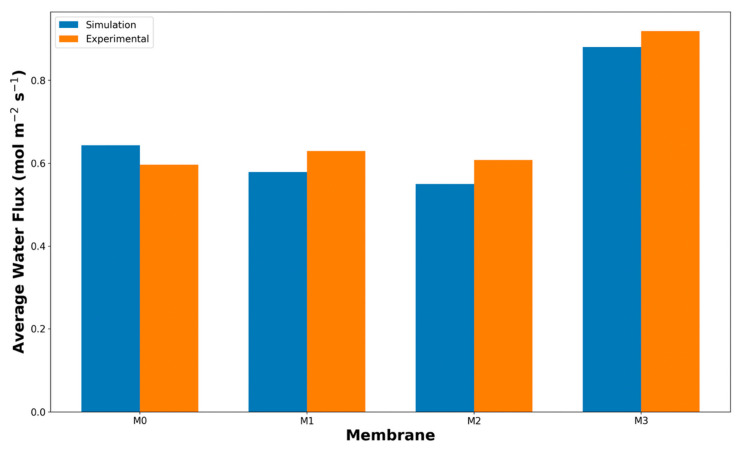
Comparison of simulation and experimental average water flux for different membranes.

To assess the reliability of the developed numerical model, the simulated values of salt rejection and water flux were compared with the experimental results obtained for the fabricated TFC membranes under the same operating conditions (0.56 m s^−1^ and 2000 ppm NaCl feed solution) [19]. The experimental values used for validation were averages obtained from repeated membrane performance measurements under identical operating conditions, whereas the simulated values represent deterministic outputs from the numerical model. Since salt rejection and water flux were evaluated at multiple positions along the membrane length, the simulated values were averaged to obtain the overall predicted performance for each membrane. Figure 3 and Figure 4 compare the mean experimental data with the corresponding averaged simulation results for salt rejection and water flux of the pristine and GO–PAMAM-modified membranes (M0–M3).

The agreement between the simulated and experimental results was quantified using the mean relative error (*MRE*). The obtained *MRE* values for water flux and salt rejection were found to be 7.41% and 1.23%, respectively, indicating that the model predictions are in good agreement with the experimental observations. This confirmed that the developed COMSOL model could reasonably predict the separation performance of the fabricated membranes under the investigated conditions.

The *MRE* was calculated using the following expression, written as Equation (11):

(11) $$MRE=\frac{1}{n}\sum_{i=1}^{n}\left(\frac{\left|True\;Value_{i}-Predicted\;Value_{i}\right|}{\left|True\;Value_{i}\right|}\right)\times 100\;$$

where *n* represents the total number of observations. The true value corresponds to the experimentally measured data, whereas the predicted value was obtained from the simulation.

## 5. Results and Discussion

CFD analysis was performed to evaluate the RO performance of the experimentally fabricated TFC membranes prepared using GO–PAMAM-modified PSF substrates. The simulation and solution of the governing equations were carried out using COMSOL Multiphysics. The effect of GO–PAMAM incorporation into the PSF support layer on membrane performance was investigated in this study. The variation in water flux and solute concentration along the membrane length was analyzed using the operating parameters listed in Table 3. Finally, the numerical results were compared for different membranes (M0–M3) to assess the influence of substrate modification on desalination performance, and the membrane exhibiting the highest water flux and salt rejection was identified.

**Figure 5:**
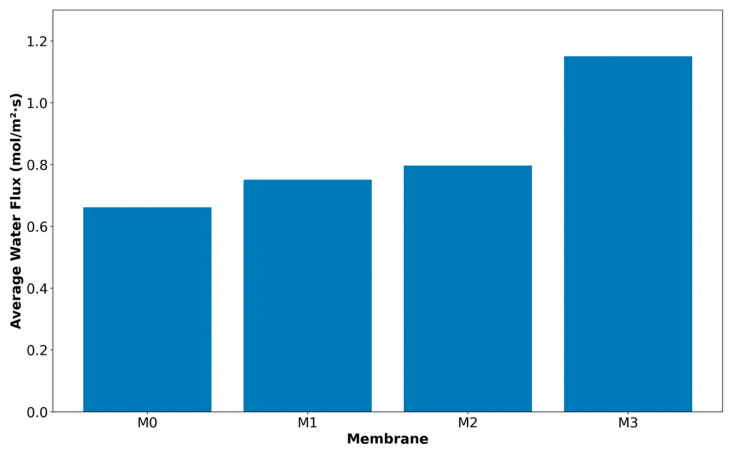
Average pure water flux of TFC membranes fabricated on GO–PAMAM-modified PSF substrates.

Figure 5 presents the experimentally measured average pure water flux of TFC membranes fabricated on PSF substrates modified with varying concentrations of GO–PAMAM, using deionized (DI) water as the feed solution. The use of DI water eliminated the influence of osmotic pressure and solute–membrane interactions, thereby enabling the intrinsic permeability of the membranes to be evaluated based on their structural characteristics. As shown in the Figure 5, variations in water flux were observed with the incorporation of GO–PAMAM into the substrate layer, indicating that substrate modification influenced the transport behavior of the resulting thin-film composite membranes.

The pristine membrane (M0) exhibited an average pure water flux of 0.6617 mol m^−2^ s^−1^, while membranes M1 and M2 showed flux values of 0.7513 and 0.7964 mol m^−2^ s^−1^, respectively. The M3 membrane demonstrated an average flux of 1.1504 mol m^−2^ s^−1^. The increase in water permeation with GO–PAMAM incorporation can be explained by the combined effects of improved hydrophilicity and modified substrate morphology. GO contains oxygen-containing functional groups, while PAMAM provides amine and amide groups; these hydrophilic groups increased the affinity of the membrane to water molecules. During phase inversion, the presence of GO–PAMAM could also accelerate solvent–non-solvent exchange, promoting the formation of more favorable water transport pathways within the PSF support layer. Therefore, at appropriate loading, GO–PAMAM incorporation can enhance water permeation. However, this effect is loading-dependent, as excessive nanocomposite content may lead to aggregation or less uniform pore formation, which can reduce the overall permeability–selectivity balance.

These results indicate that different concentrations of GO–PAMAM incorporated into the PSF substrate have a measurable effect on the pure water permeation characteristics of the fabricated TFC membranes.

**Figure 6:**
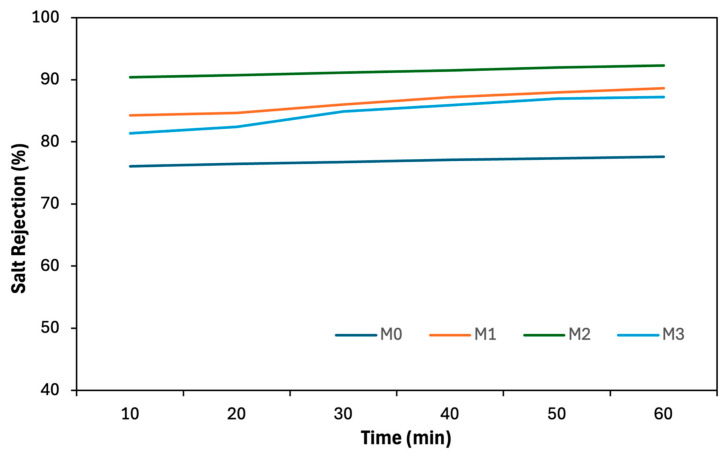
Salt rejection of TFC membranes (M0–M3) as a function of filtration time at a feed concentration of 2000 ppm NaCl.

Prior to salt rejection measurements, all membranes were initially compacted using DI water to ensure stabilization of the membrane structure under the applied operating pressure, and average values are shown in Figure 5. Subsequently, an aqueous NaCl solution with a feed concentration of 2000 ppm (34.2 mol m^−3^) was introduced into the system to evaluate the desalination performance of the fabricated TFC membranes. The time-dependent salt rejection behavior of membranes M0–M3 is presented in Figure 6. As shown in Figure 6, the salt rejection values for membranes M0 and M2 remained relatively stable over the entire testing duration, exhibiting minimal fluctuation with time. In contrast, membranes M1 and M3 showed a gradual increase in rejection values during the initial stages of operation, particularly after approximately 20 min of filtration. This variation in rejection behavior suggests that the incorporation of different concentrations of GO–PAMAM into the PSF substrate influenced the temporal response of the membrane during desalination. The temporal variation in salt rejection may be associated with membrane compaction, progressive wetting of the substrate and PA selective layer, and stabilization of water and salt transport pathways during the initial filtration period. GO–PAMAM incorporation modified the hydrophilicity and morphology of the PSF substrate, which could influence pore wetting, water uptake, and the interaction between the support and the PA selective layer. In addition, the modified substrate could affect MPD uptake and distribution during interfacial polymerization, thereby influencing the uniformity and compactness of the PA layer. Among the investigated membranes, M2 containing 0.06 wt% GO–PAMAM showed the most favorable salt rejection behavior, indicating that this loading provided an optimum balance between improved substrate wettability, controlled support morphology, and PA selective layer formation. At higher loading, possible nanocomposite aggregation or non-uniform monomer distribution may disturb PA layer formation, leading to less stable salt permeation behavior.

The average salt rejection values obtained for membranes M0, M1, M2, and M3 were 76.87%, 86.44%, 91.34%, and 84.78%, respectively, indicating that different levels of GO–PAMAM incorporation into the substrate had a measurable effect on the salt separation performance of the resulting TFC membranes.

**Figure 7:**
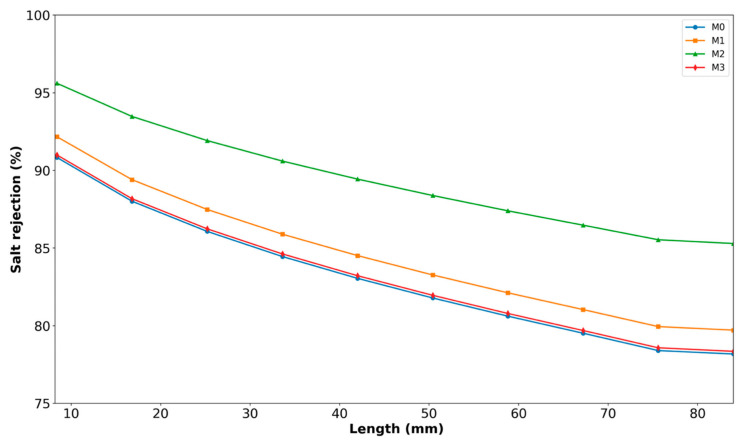
Simulated salt rejection of TFC membranes (M0–M3) along the membrane length at a feed concentration of 2000 ppm NaCl.

In addition to the experimentally evaluated salt rejection performance, numerical simulations were used to investigate the spatial variation of salt rejection along the membrane length. Figure 7 presents the simulated salt rejection profiles for membranes M0–M3 as a function of membrane length under the operating conditions listed in Table 3. As shown in the Figure 7, a gradual decrease in salt rejection was observed along the flow direction for all membranes. This decrease was mainly related to the gradual increase in local permeate-side salt concentration along the membrane length. Since salt rejection was calculated from the ratio of permeate concentration to feed concentration, an increase in permeate-side concentration resulted in a lower local rejection value. The increase in permeate concentration occurred because salt continuously passed through the semi-permeable membrane interface according to the assigned salt permeability coefficient.

Among the investigated membranes, M2 exhibited consistently higher salt rejection values along the entire membrane length compared to the other membranes, while M0 showed the lowest rejection throughout the channel. Membranes M1 and M3 demonstrated intermediate rejection behavior, with rejection values decreasing steadily from the inlet to the outlet of the membrane module. Such spatial reduction in salt rejection could influence the overall quality of the permeate water and affect the operational efficiency of the desalination system, as increased salt passage could result in elevated solute concentration in the treated water. These simulation results indicate that the incorporation of different concentrations of GO–PAMAM into the PSF substrate influenced the predicted salt transport characteristics of the resulting TFC membranes along the membrane length.

### 5.1. Simulation of Solute Concentration Distribution Along the Membrane Length

**Figure 8:**
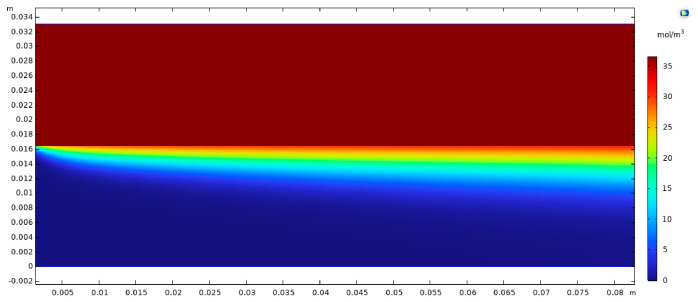
Simulated solute concentration distribution in the feed and permeate channels for the pristine membrane (M0).

**Figure 9:**
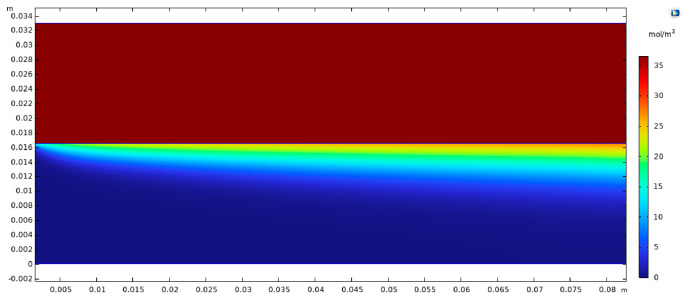
Simulated solute concentration distribution in the feed and permeate channels for the modified GO-PAMAM membrane (M2).

The distribution of NaCl concentration on both the feed and permeate sides of the membrane provides direct insight into the predicted separation behavior of the numerically modeled RO module. In the present work, the solute concentration fields were evaluated as part of the COMSOL-based investigation to examine how substrate modification (GO–PAMAM incorporation) influenced salt transport across the membrane boundary. Figure 8 and Figure 9 present the simulated NaCl concentration distributions for membranes M0 and M2, respectively, where the upper channel corresponds to the feed side and the lower channel corresponds to the permeate side.

In both figures, the inlet feed concentration was specified as 34.2 mol m^−3^ to replicate the experimental feed condition of 2000 ppm NaCl. Under this imposed boundary condition, the feed-side concentration remained relatively uniform along the membrane length in the numerical domain. In contrast, the permeate-side concentration remained substantially lower than the feed side but gradually varied along the membrane length, indicating salt passage through the semi-permeable membrane boundary. Since the membrane was represented as an internal semi-permeable interface rather than a finite-thickness solid domain, the concentration gradient through the actual membrane thickness was not directly resolved. Therefore, the concentration distribution shown in Figure 8 and Figure 9 should be interpreted mainly as the solute concentration variation in permeate channel along the membrane length. The concentration difference between the feed and permeate sides adjacent to the membrane interface provided the driving force for salt transport across the semi-permeable boundary, consistent with the salt permeability coefficient, B, used in the solution–diffusion formulation.

A quantitative comparison of the permeate-side concentration further highlights the difference between the pristine and modified membranes. For M0, the predicted surface-averaged permeate concentration was 7.469 mol m^−3^, whereas for M2, it decreased to 4.375 mol m^−3^. This reduction in permeate concentration is consistent with the simulated salt rejection values, which increased from 78.16% (M0) to 90.08% (M2). In addition to changes in solute transport, the numerical model also predicted differences in water permeation. The simulated average water flux for M0 was 0.6436 mol m^−2^ s^−1^, while M2 exhibited a lower flux of 0.3937 mol m^−2^ s^−1^, corresponding to a 38.8% decrease in water flux relative to M0.

**Figure 10:**
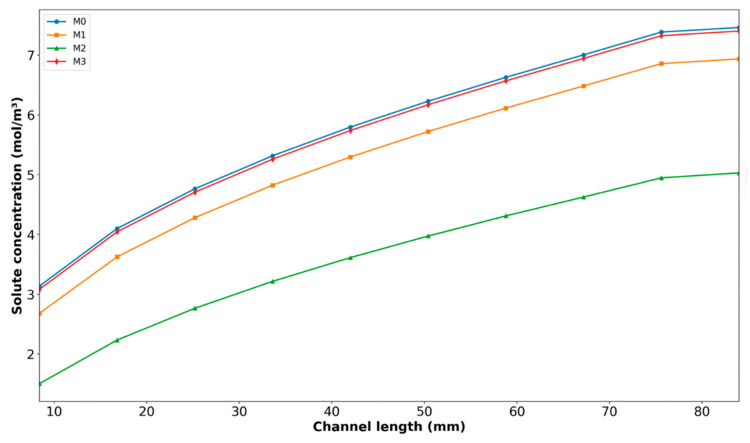
Simulated surface-averaged permeate solute concentration of TFC membranes (M0–M3) along the membrane length.

Overall, Figure 8, Figure 9 and Figure 10 show that the GO–PAMAM-modified substrate case (M2) is associated with a lower predicted permeate-side solute concentration and higher predicted salt rejection compared to the pristine membrane (M0), while simultaneously exhibiting a lower predicted water flux. These results demonstrate that substrate modification influenced both the water and salt transport characteristics captured by the numerical model through the assigned transport properties and boundary conditions.

### 5.2. Velocity Profile Along the Length of Membranes

**Figure 11:**
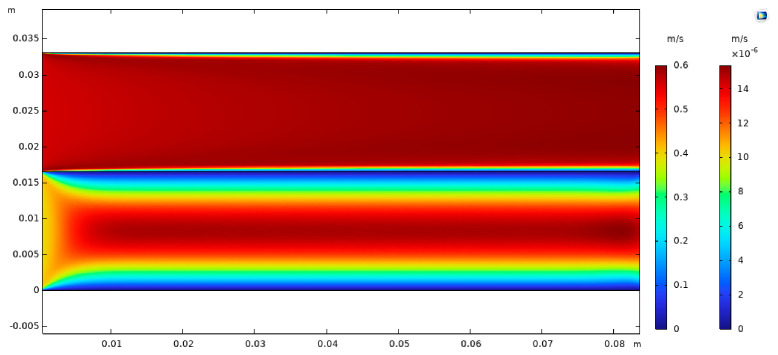
Simulated velocity distribution in the feed and permeate channels for the M2 membrane case.

The velocity distribution within the feed and permeate channels was evaluated to examine the hydrodynamic behavior of the modeled RO module. In the numerical model, the inlet velocities in both the upper (feed) and lower (permeate) channels were specified as constant according to the defined boundary conditions. Figure 11 presents the simulated velocity contours in the feed and permeate channels for the M2 membrane case, which was selected as a representative case because it exhibited the highest salt rejection among the investigated membranes.

It can be observed that after a short entrance region from the channel inlet, the flow developed into a fully established laminar velocity profile along the channel length. The maximum fluid velocity occurred at the central region of the channels and gradually decreased toward the channel walls.

**Figure 12:**
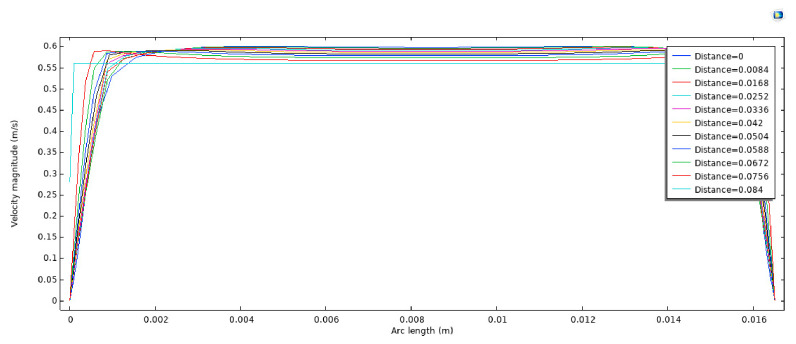
Velocity magnitude profile of feed channel across the channel height at different axial positions along the membrane length for membrane M2.

**Figure 13:**
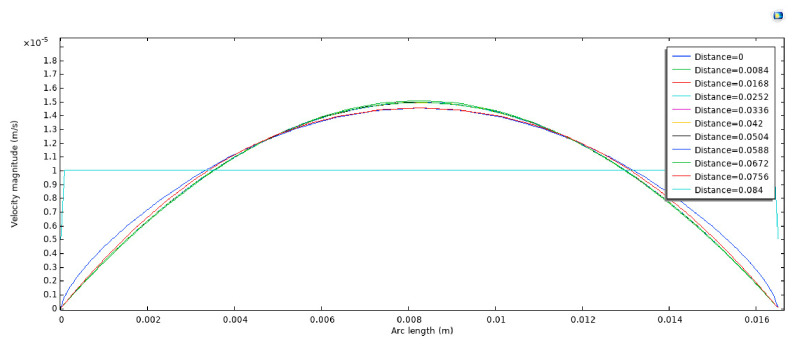
Velocity magnitude profile across the permeate channel at different axial positions along the membrane length for membrane M2.

In the vicinity of the membrane surfaces and channel walls, the velocity approached zero due to the imposed no-slip boundary condition at the solid–fluid interface. This behavior is characteristic of laminar flow within confined channels. Figure 12 and Figure 13 illustrate the velocity distribution across the feed and permeate channels, where an increase in velocity toward the center of the channels was evident.

### 5.3. Effect of GO-PAMAM on Membrane Transport Performance

**Figure 14:**
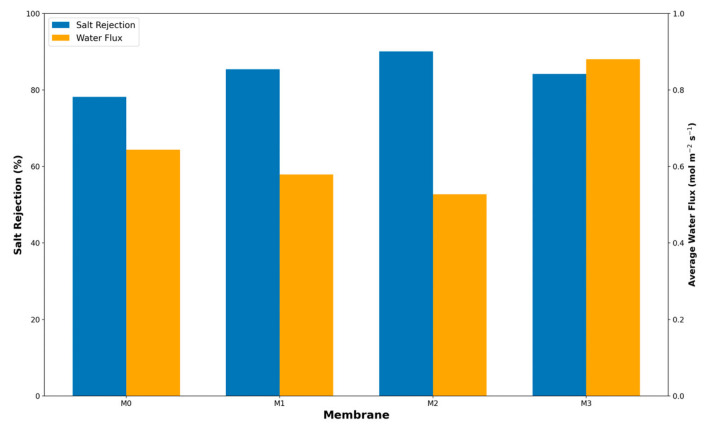
Comparison of simulated salt rejection and average water flux for TFC membranes M0–M3.

The incorporation of GO–PAMAM into the PSF substrate was found to influence both water and salt transport characteristics of the resulting TFC membranes. Although GO–PAMAM introduces hydrophilic functional groups into the substrate, the overall membrane performance was also governed by PA selective layer formation, salt permeability, and the permeability–selectivity balance. As observed from the experimental results reported in our previous study [19] and the numerical results obtained in the present work, membrane M2 showed the most favorable transport performance, with improved salt rejection while maintaining acceptable water flux relative to the pristine membrane (M0). This behavior indicates that optimized GO–PAMAM loading improves selective transport rather than simply increasing water permeation. As shown in Figure 14, the simulated results for M0–M3 demonstrate that M2 provided the highest salt rejection, while the variation in water flux reflects the transport trade-off associated with different GO–PAMAM loadings.

## 6. Conclusions

Computational fluid dynamics provides a means to resolve spatial transport phenomena in reverse osmosis membranes that are not accessible through bulk experimental measurements. A two-dimensional model was developed in COMSOL Multiphysics by coupling the Laminar Flow and Transport of Diluted Species interfaces, while membrane transport was described using a solution–diffusion formulation parameterized by experimentally determined intrinsic permeability coefficients for membranes incorporating graphene oxide–poly(amidoamine) (GO–PAMAM) within the polysulfone substrate.

The simulations reproduced the experimentally observed trend, with an intermediate GO–PAMAM loading (0.06 wt%) yielding the highest salt rejection, increasing from 78.16% for the pristine membrane to 90.08% for the modified membrane. This improvement was accompanied by a reduction in surface-averaged permeate concentration. In addition to overall performance, the model captured a progressive decrease in salt rejection along the membrane length for all membranes, indicating spatial variation in transport behavior under crossflow conditions. Such variations were not directly accessible from conventional measurements based on bulk averages and could influence permeate quality along the module.

Model predictions showed good agreement with experimental data, with mean relative errors of 1.23% for salt rejection and 7.41% for water flux. These results indicate that the solution–diffusion framework, when parameterized using experimentally derived permeability coefficients, can capture the transport behavior of GO–PAMAM-modified membranes. The present approach provides a basis for linking substrate modification to membrane performance and offers a framework for evaluating support-engineered RO membranes under operating conditions.

## Figures and Tables

**Table 1:** Constructive materials and their specifications for TFC membrane fabrication.

Material	Specifications	Role and Application
Polysulfone (PSF)	Mw ≈ 35 kDa, Sigma-Aldrich, St. Louis, MO, USA	Base polymer for fabrication of porous support layer
Nonwoven polyester fabric	97 ± 10 µm thickness, Wellspring, Gwangju City, South Korea	Mechanical backing support for TFC membrane
*N,N*-Dimethylformamide (DMF)	≥99.9%, Sigma-Aldrich, St. Louis, MO, USA	Solvent for PSF and GO–PAMAM dispersion during phase inversion
Graphene oxide (GO)	Synthesized via modified Hummers’ method	Nanomaterial precursor for substrate modification
Poly(amidoamine) dendrimer (PAMAM G2)	Second generation, 99.9%, Dendritech Inc., Midland, MI, USA	Functionalizing agent to enhance hydrophilicity and compatibility
Graphene oxide–PAMAM (GO–PAMAM)	Synthesized nanocomposite	Substrate modifier to regulate pore formation and PA layer development
*m*-Phenylenediamine (MPD)	≥99%, Sigma-Aldrich, MO, USA	Aqueous-phase monomer for polyamide selective layer formation
Trimesoyl chloride (TMC)	≥98%; TCI, Tokyo, Japan	Organic-phase monomer for polyamide selective layer formation
*n*-Hexane	Reagent-grade	Organic solvent for TMC during interfacial polymerization
Sodium dodecyl sulfate (SDS)	Reagent-grade	Surfactant to improve substrate wetting and MPD distribution
Distilled/deionized water	Laboratory-grade	Non-solvent for phase inversion and membrane washing

**Table 2:** Geometric dimensions, feed operating conditions, and transport parameters used in the numerical simulation of the RO module.

Parameter	Value
Membrane surface area (cm^2^)	32.76
Membrane length (cm)	8.4
Feed flow velocity (m s^−1^)	0.56
Feed NaCl concentration (mol m^−3^)	34.2
Inlet flow velocity (m s^−1^)	0.00001
Inlet flow concentration (mol m^−3^)	0.001

**Table 3:** Intrinsic salt permeability coefficients (B) assigned to the membrane interface in the numerical model for TFC membranes with varying GO–PAMAM loadings.

Membrane Type	GO-PAMAM wt%	Salt Permeability, B (m/s)
M0	0	3.236 × 10^−6^
M1	0.03	1.777 × 10^−6^
M2	0.06	1.039 × 10^−6^
M3	0.10	2.979 × 10^−6^

## Data Availability

The original contributions presented in this study are included in the article. Further inquiries can be directed to the corresponding author.

## Nomenclature and Abbreviations

A	Water permeability coefficient (m s^−1^ Pa^−1^)
B	Salt permeability coefficient (m s^−1^)
C	Solute concentration (mol m^−3^)
C_f_	Feed concentration (mol m^−3^)
C_p_	Permeate concentration (mol m^−3^)
D	Diffusion coefficient (m^2^ s^−1^)
J_w_	Water flux (mol m^−2^ s^−1^)
J_s_	Solute flux (mol m^−2^ s^−1^)
n	Outward normal vector
P	Hydraulic pressure (Pa)
R	Salt rejection (%)
Re	Reynolds number
U	Velocity vector (m s^−1^)
ρ	Density of solution (kg m^−3^)
μ	Dynamic viscosity (Pa s)
DMF	Dimethylformamide
SEM	Scanning electron microscopy
GO	Graphene oxide
PAMAM	Poly(amidoamine)
IP	Interfacial polymerization
LMH	Liter per square meter per hour (L m^−2^ h^−1^)
MPD	m-Phenylenediamine
NaCl	Sodium chloride
PA	Polyamide
PSF	Polysulfone
RO	Reverse osmosis
TFC	Thin-film composite
TMC	Trimesoyl chloride
XRD	X-ray diffraction|
FEM	Finite element method
CFD	Computational fluid dynamics
TDS	Transport of diluted species
DI	Deionized water
IP	Interfacial polymerization
MRE	Mean relative error
